# Genome-Wide Identification and Analysis of the Perennial Ryegrass (*Lolium perenne* L.) WRKY Family Associated with Heat Stress

**DOI:** 10.3390/ijms27209332

**Published:** 2026-10-20

**Authors:** Wei Chen, Nan Song, Jing Zhang, Bin Xu, Yingjun Chi

**Affiliations:** 1 College of Agro-Grassland Science, Nanjing Agricultural University, Nanjing 210095, China; 2 College of Landscape Architecture, Jiangsu Vocational College of Agriculture and Forestry, Jurong 212400, China

**Keywords:** gene family, heat stress, perennial ryegrass, WRKY transcription factors

## Abstract

Perennial ryegrass, the most widely cultivated forage and turfgrass, exhibits a relatively poor temperature tolerance, which restricts its distribution and utilization in warmer regions. WRKY transcription factors regulate diverse biological processes in plants, including development and stress responses. Here, we report the comprehensive identification of 97 *WRKY* genes (*LpWRKYs*) in perennial ryegrass. The *LpWRKYs* were unevenly distributed across seven chromosomes, with one gene anchored to a scaffold. These genes were classified into three major groups and five subgroups based on phylogenetic relationships and structural domain diversity. All LpWRKYs contained either one or two WRKY domains and the LpWRKYs within the same group had similar motif compositions. A total of 12 tandem duplicates and 27 segmental duplicates were identified, suggesting that gene duplication might have contributed to the expansion of the *LpWRKY* gene family. Ka/Ks analysis revealed that all duplicated *LpWRKY* gene pairs were subjected to purifying selection during the evolution. Co-linearity analysis showed strong co-linearity between *LpWRKYs* and *OsWRKYs* from rice. *Cis*-acting elements in the *LpWRKY* promoters were related to growth and development, phytohormone responses, and stress responses. RNA-seq and qRT-PCR analysis revealed that the expression levels of *LpWRKY5/16/33/35* were significantly up-regulated, while *LpWRKY21*/*92* were significantly down-regulated in roots under heat stress. Additionally, *LpWRKY12/21/33/35/43/89/92/96* displayed significant down-regulation in leaves under heat stress. It was suggested that these *LpWRKYs* were potentially associated with the heat-stress response in perennial ryegrass. Notably, *LpWRKY33* and *LpWRKY35* exhibited distinct tissue-specific transcriptional patterns in response to heat stress, implying their participation in complex tissue-dependent regulatory networks. Protein interaction prediction further suggested that LpWRKY96 may function as a potential central regulator in the heat response signaling network. Taken together, this study provides clues into the regulatory roles of the WRKY gene family in perennial ryegrass.

## 1. Introduction

Perennial ryegrass (*Lolium perenne* L.) is the most widely cultivated perennial cool-season forage and turf grass, distinguished by its high forage quality, rapid establishment, and excellent turf performance. Native to Europe, Asia and northern Africa, it has been introduced and extensively planted in subtropical and temperate regions globally. As a cool-season grass, perennial ryegrass exhibits a relatively narrow temperature tolerance range, which restricts its distribution and utilization in warmer regions. When temperatures exceed 30–35 °C, its growth and development are significantly inhibited. With the intensification of global climate change, the frequency and intensity of high-temperature events are increasing, leading to issues such as stunted growth, reduced forage yield and deteriorated turf quality. Consequently, systematically investigating the heat tolerance mechanisms of perennial ryegrass and developing strategies to improve its heat tolerance have become crucial research areas in the field of grassland science and turf management.

WRKY transcription factors (TFs) are one of the largest families of regulatory proteins in plants, playing roles in diverse plant processes including growth, development, responses to biotic and abiotic stress, and plant innate immunity, such as microbe- or pathogen-associated molecular pattern-triggered immunity (MTI or PTI) and effector-triggered immunity (ETI) [1]. WRKY TFs typically contain a highly conserved WRKY domain at the C-terminus, harboring the WRKYGQK heptapeptide sequence and a C2H2 or C2HC-type zinc finger structural motif [2]. Phylogenetically, WRKY proteins are categorized into three major groups: group I, II and III [3]. Group II WRKY proteins can be further subdivided into five subgroups: IIa, IIb, IIc, IId, and IIe [4]. WRKY proteins positively or negatively regulate gene expression by binding to the *cis*-acting element W-box (TTGACC/T) in the promoter region of target genes [5,6]. Despite their diverse biological functions, most analyzed WRKY proteins recognize W-box sequences [7]. Additionally, WRKY TFs physically interact with a wide range of proteins with roles in signaling, transcription and chromatin remodeling, which is necessary for establishing the complex regulatory and functional network of WRKY TFs [7]. The protein-protein interactions of WRKY TFs could influence their DNA-binding and transcription-regulatory activities, thereby controlling or modulating WRKY-regulated gene expression.

Since the first WRKY gene, *SPF1*, was identified in sweet potato (*Ipomoea batatas*) [8], numerous members of the *WRKY* gene family have been identified across various plant species, including 67 from *Leonurus japonicus* [9], 176 *WRKY* genes from elephant grass [10], 242 from *Camelina sativa* [11], 94 from *Sorghum bicolor* [12], 83 from tomatoes, 120 from Zea *mays* [13], 145 from white clover (*Trifolium repens*) [14], 59 from red clover (*Trifolium pratense* L.) [15], and 91 from alfalfa (*Medicago sativa*) [16]. WRKY homologs have been extensively documented to play transcriptional regulatory roles in adaptations to various stress environments across many species. An increasing number of studies demonstrate that the WRKY family is involved in the high-temperature response and help plants to adapt the temperature changes. For example, *AtWRKY25/26/33* have been shown to positively regulate heat tolerance [17,18]. Constitutive overexpression of *AtWRKY25* and *AtWRKY26* enhanced resistance to heat stress [17]. The group II WRKY protein AtWRKY39 was induced by heat treatment, and was positively regulated by the salicylic acid (SA) and jasmonic acid (JA) signaling pathways. Overexpression of *AtWRKY39* confered enhanced thermotolerance in *Arabidopsis* [19]. *TaWRKY70* was positively involved in high-temperature seedling plant (HTSP) resistance to *Puccinia striiformis* f. sp. *tritici* (Pst), which induces stripe rust in wheat (*Triticum aestivum*) [20]. *TaWRKY33* from WRKY Group II was involved in responses to high-temperature, low-temperature, abscisic acid (ABA) and jasmonic acid methylester (MeJA). Transgenic *Arabidopsis* overexpressing *TaWRKY33* exhibited enhanced tolerance to heat stress [21]. A group I WRKY transcription factor in tomato, SlWRKY3, confered thermotolerance by activating the expression of abiotic stress-responsive genes involved in reactive oxygen species (ROS) scavenging, such as the *SlGRXS1* gene cluster [22]. The MdHSC70-MdWRKY75 module mediated basal apple thermotolerance by regulating the expression of heat shock factor genes [23]. StWRKY75 acts as a positive regulator of potato thermotolerance by enhancing growth, antioxidant capacity, proline metabolism, photosynthesis, and heat-responsive gene expression [24]. However, whether and how WRKY TFs in perennial ryegrass are involved in the response to heat stress remains rarely investigated.

Based on previous findings, we hypothesize that WRKY TFs may mediate the regulation of stress resistance in perennial ryegrass, despite the absence of genome-wide detection of this gene family in ryegrass. Utilizing integrated genomic and transcriptomic resources of perennial ryegrass, we performed a systematic genome-wide characterization of WRKY TFs, coupled with subcellular localization prediction. A comprehensive analysis was performed to examine their gene structures, chromosomal locations, conserved motifs, phylogenetic tree, and *cis*-acting elements within the WRKY gene family. Furthermore, the expression profiles of *WRKY* genes were extensively detected using RNA-Seq data, and a selected set of *LpWRKY* gene transcripts were validated by quantitative real-time RT-PCR (qRT-PCR) in ryegrass seedlings under stress conditions. Our study offers new scientific resources for understanding the biological roles of WRKYs underlying strong stress tolerance in ryegrass, providing valuable information for elucidating the molecular mechanisms and facilitating the genetic improvement of this cool-season grass and other crops.

## 2. Results

### 2.1. Identification of 97 WRKYs in the Perennial Ryegrass Genome

To systematically identify WRKY proteins encoded in the perennial ryegrass genome, 72 *Arabidopsis thaliana* and 102 rice WRKY protein sequences were used as queries to search the publicly available genome sequences of perennial ryegrass by BLAST (Basic Local Alignment Search Tool, https://blast.ncbi.nlm.nih.gov/Blast.cgi, accessed on 25 August 2026). All candidate sequences were further validated using a Hidden Markov Model (HMM) with the WRKY-domain (PF03106), which is characterized by a conserved N-terminal WRKYGQK motif and a unique zinc-finger structural feature. A total of 97 putative WRKY-encoding genes were identified and annotated in perennial ryegrass (Table 1). Based on chromosomal positions retrieved from the perennial ryegrass genome database, the *LpWRKY* genes were systematically renamed from *LpWRKY1* to *LpWRKY97*, and their characteristics were further specified, including chromosomal location, protein length, molecular weight (MW), theoretical isoelectric point (pI), phylogenetic classification and other genetic features (Table 1 and Table S1).

The LpWRKY proteins exhibited wide variations in sequence length and physicochemical properties across the family.The proteins length ranged from 138 amino acids (LpWRKY61) to 1256 amino acids (LpWRKY57), reflecting a broad and uneven distribution. Accordingly, their MWs varied from 14.88 kDa (LpWRKY61) to 141.44 kDa (LpWRKY57), while their pI values ranged from 4.91 (LpWRKY41) to 10.15 (LpWRKY29), with 58 LpWRKY proteins being acidic (pI <  7.0) (Table 1 and Table S1). The average protein length, MW and pI of the LpWRKY family were 372 amino acids, 40.14 kDa and 7.01, respectively. Notably, all LpWRKY proteins yielded negative grand average of hydropathicity values, indicating that they are all hydrophilic proteins (Table S1). Protein stability prediction indicated that nearly all LpWRKYs were unstable proteins, whereas only three members (LpWRKY16, LpWRKY25, LpWRKY81) with an instability index below 40 were considered to be stable proteins. Additionally, most LpWRKY proteins were localized in the nucleus, except for 7 LpWRKYs in chloroplast, 3 LpWRKYs in cytoplasm, 2 LpWRKYs in mitochondrion and 1 LpWRKYs in peroxisome (Table 1).

### 2.2. Alternative Splicing Occurring Among LpWRKY Genes

As a critical post-transcriptional regulatory mechanism in eukaryotes, alternative splicing (AS) causes a single gene locus to generate two or more open reading frames (ORFs) with distinct transcription start/end sites and splice junctions. Such alternatively processed ORFs consequently drive the biosynthesis of distinct protein isoforms derived from identical genetic loci. In this study, multiple annotated transcript isoforms corresponding to eight *LpWRKY* genes were extracted from the NCBI genome annotation of perennial ryegrass, yielding 17 distinct transcript variants. (Table S2). All these splice variants correspond to annotated mature mRNA sequences of perennial ryegrass. For example, the gene loci LOC127319033, annotated as *LpWRKY7* in this study, generated two mature spliced mRNA transcripts (XM_051349287.2 and XM_051349288.2).

### 2.3. Phylogenetic Relationship of WRKY Proteins

**Figure 1:**
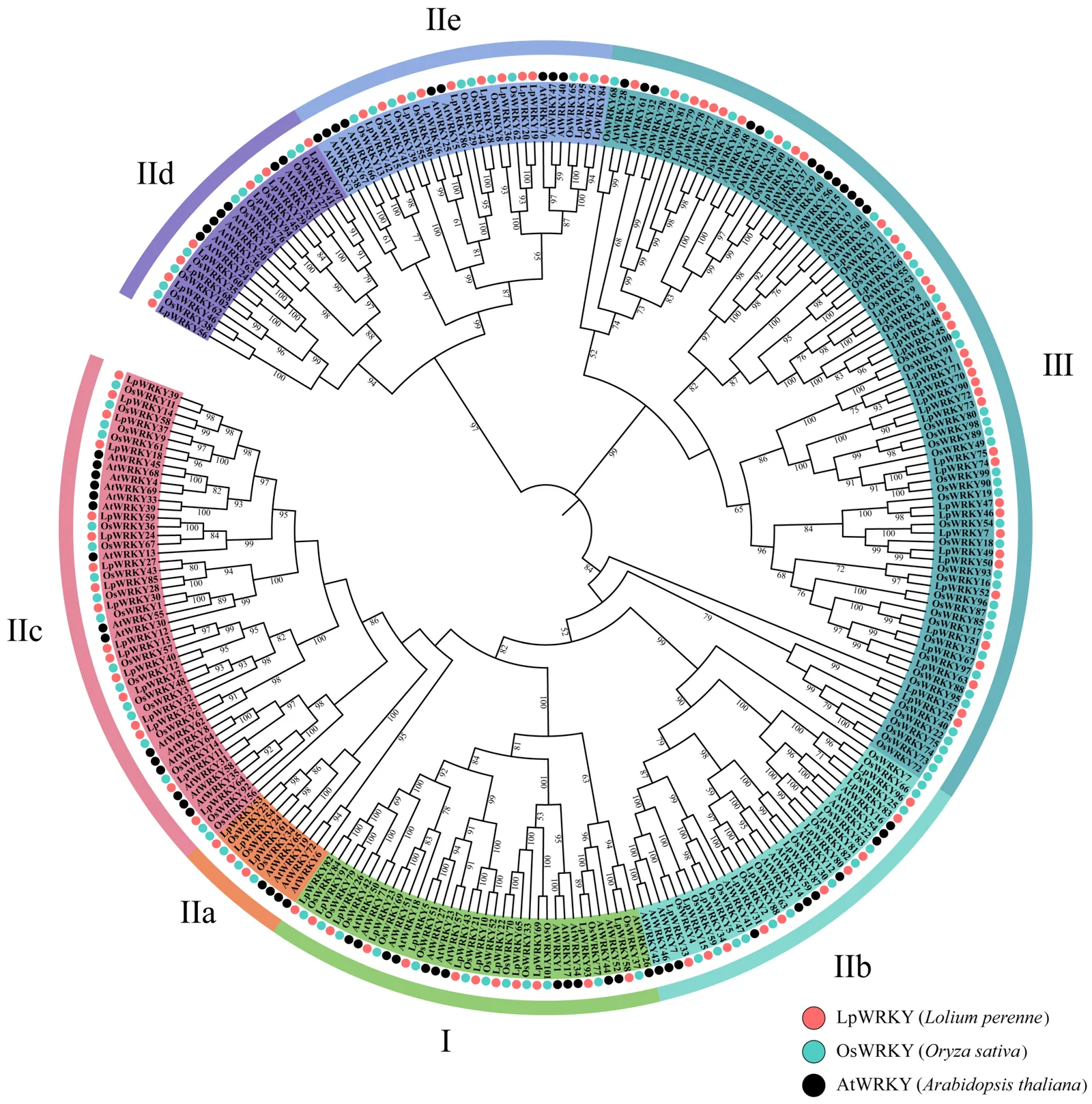
Phylogenetic analysis of WRKY gene family in perennial ryegrass, rice and *Arabidopsis*. A maximum likelihood phylogenetic tree was constructed based on the amino acid sequences of LpWRKYs, OsWRKYs and AtWRKYs using FastTree v2.1.10. Seven groups are marked with different colors.

To investigate the evolutionary relationships and facilitate subfamily classification of the LpWRKY members, a maximum-likelihood phylogenetic tree was built based on multiple sequence alignment of the full-length sequences of 97 LpWRKYs proteins from perennial ryegrass, 102 OsWRKYs proteins from rice and 72 AtWRKYs from *Arabidopsis* (Figure 1). The phylogenetic analysis revealed that all 97 LpWRKY proteins were categorized into three major groups: group I, II and III, based on the classification described by a previous report on rice [25], indicating an evolutionary conservation between these two species. Group I contained 12 members, group II contained 48 members, and group III contained 37 members (Figure 1 and Table 1). Group II has the largest member number, which can be further categorized into five distinct subgroups: II-a, II-b, II-c, II-d and II-e containing 3, 10, 17, 7 and 11 members of LpWRKYs respectively.

### 2.4. Conserved Motifs and Gene Structural Analysis of LpWRKYs

**Figure 2:**
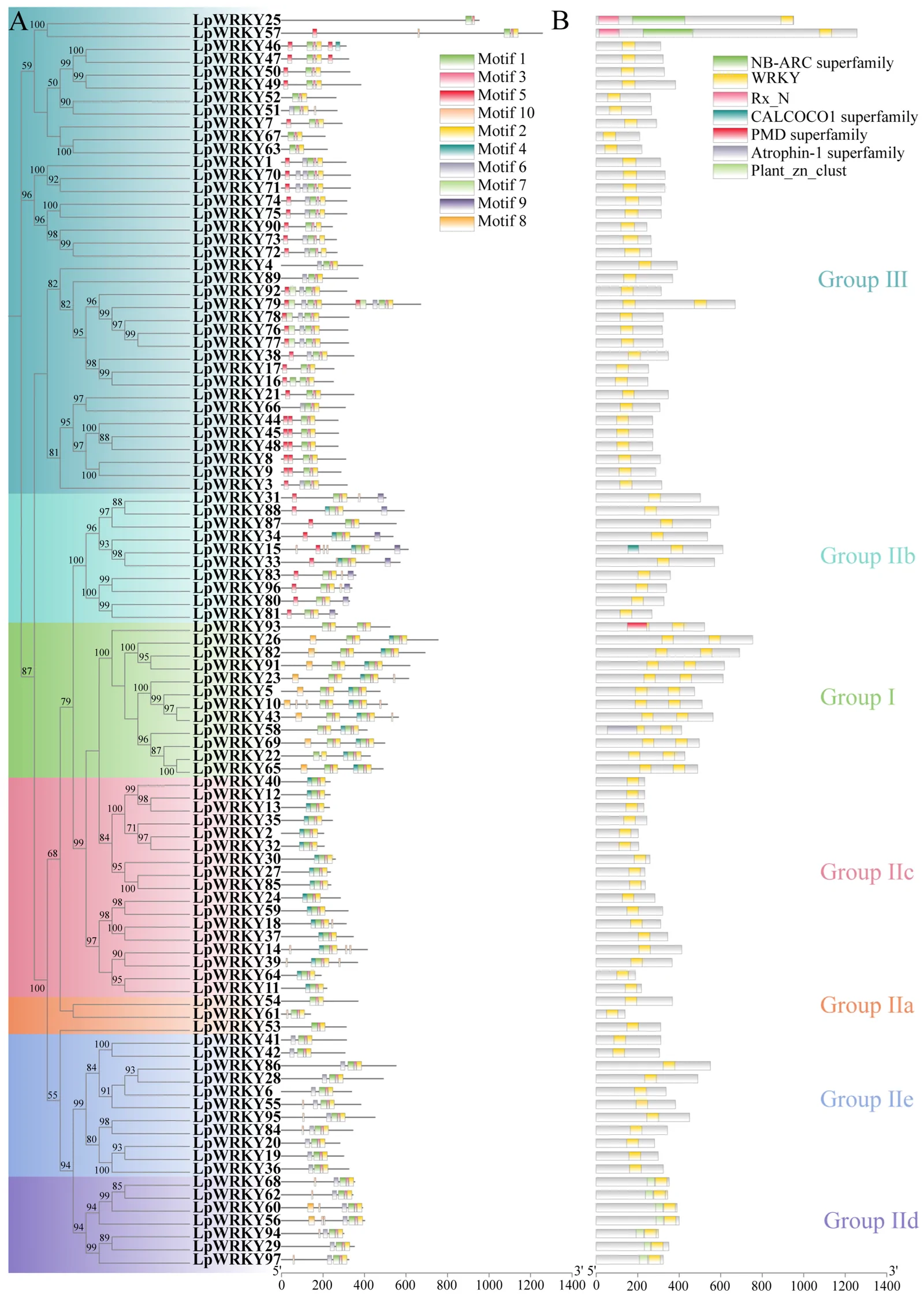
Conserved motif and conserved domain of the WRKY gene family in perennial ryegrass. (**A**) The phylogenetic tree (**left**) constructed based on full-length amino acid sequences, and the distribution of conserved motifs (**right**) identifed by MEME analysis. Motifs 1 to 10 are represented by differently colored boxes. The scale bar below indicates the length of the amino acid sequence. (**B**) Conserved domain architecture annotated using Pfam and CDD databases. Color-coded boxes represent distinct domain types.

To gain insight into the functional regions and structural characteristics of LpWRKY proteins, the MEME (Multiple Em for Motif Elicitation) web tool was employed to reveal the conserved motifs among all 97 LpWRKY proteins. A total of 10 distinct conserved motifs were identified, namely Motifs 1–10 (Figure 2). These 10 conserved motifs are indicated with coloured boxes according to their scale, with sizes ranging from 8 to 29 aa residues (Table S3). Among these individuals, Motif 1 corresponds to the core conserved WRKY domain and Motif 2 encodes the characteristic zinc finger structure. Each LpWRKY protein had at least three conserved motifs, with the maximum number being 12 conserved motifs (LpWRKY79). The motif distribution analysis revealed that members within the same group or subgroup exhibited similar motif composition and arrangement, indicating functional conservation within clades (Figure 2). Specifically, group I members possessed two WRKY motifs located at the N-terminal and C-terminal regions, whereas all members of the group II and group III contained only one WRKY motif (Figure 2).

**Figure 3:**
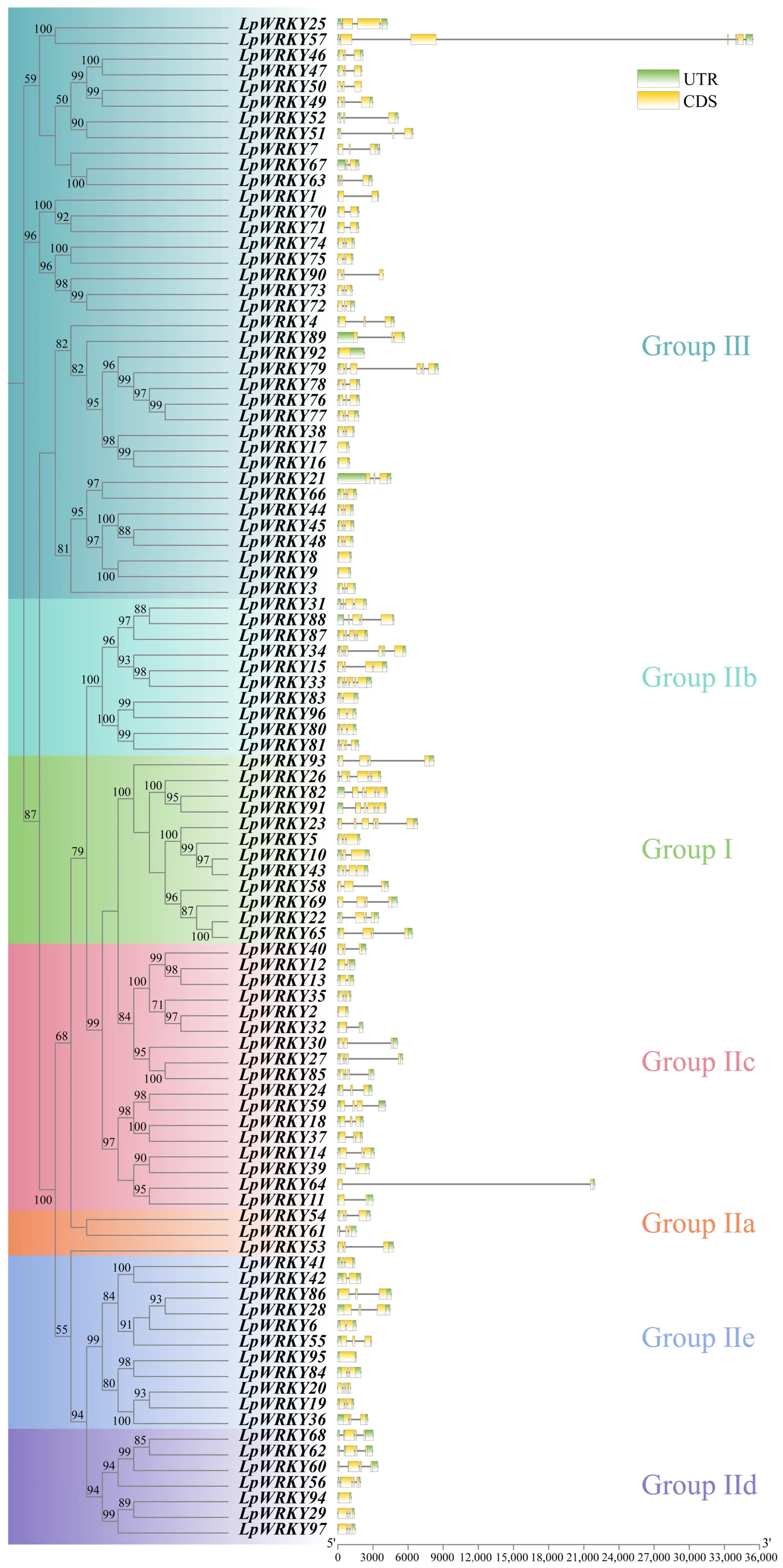
Exon-intron structure of *WRKY* gene family in perennial ryegrass by TBtools-II (v2.326). Yellow boxes represent the coding sequence (CDS), green boxes represent the non-translated region (UTR), and black lines represent introns. The scale bar below indicates the lengths of exons, introns, and UTRs.

Exon-intron structural analysis was performed by comparing genomic DNA sequences to further characterize the structural composition of *LpWRKY* genes. The number of introns in *LpWRKY* genes varied from 0 to 6, with 50 *LpWRKY* genes (51.5% of the total family) containing two introns (Figure 3). *LpWRKY57* contained the maximum intron number of six. The widespread variation in gene structures indicates frequent exon gain and loss events occurred during the evolutionary expansion of the *LpWRKY* family, which is a key driver of functional diversification among *WRKY* members in perennial ryegrass.

### 2.5. Chromosomal Distribution, Duplication and Synteny Analysis of LpWRKYs

**Figure 4:**
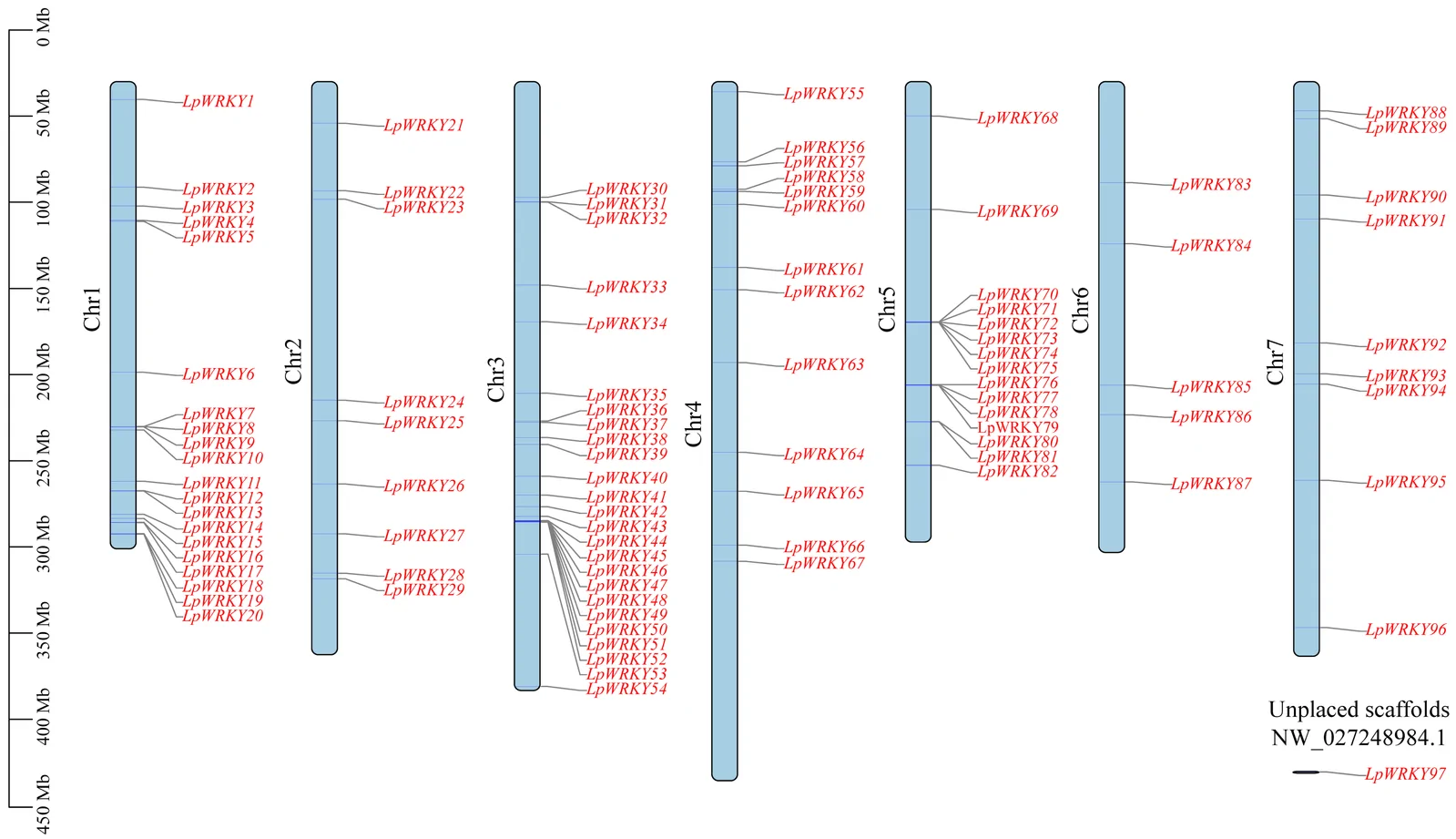
Chromosomal mapping of *WRKY* gene family members in perennial ryegrass. Vertical bars represent the 7 perennial ryegrass chromosomes, arranged in ascending order of chromosome number. The vertical axis indicates the physical length of each chromosome in megabases (Mb). Chromosome numbers are shown in black, and gene identifiers (*LpWRKY*1 to *LpWRKY*97) are highlighted in red.

The physical mapping of all 97 *LpWRKYs* on the perennial ryegrass genome revealed an uneven chromosomal distribution across all seven chromosomes (Figure 4). Notably, *LpWRKY97* could not be anchored to any chromosome since it is present on a scaffold region. The minimal number of *LpWRKY* genes (5 loci) were located on chromosome 6, whereas the largest number of *LpWRKY* genes (25 loci) were detected on chromosome 3, corresponding to 25.8% of the entire *LpWRKY* family. A few regions with a higher density of *LpWRKY* genes were observed on chromosomes 1, 3 and 5, suggesting that there might be *WRKY* gene enrichment hotspots in the perennial ryegrass genome. Interestingly, the results identified conserved sequences and physical clustering of repetitive genes belonging to the same group within the same chromosomal regions, as follows: *LpWRKY44* to *LpWRKY52* (group III) were located on chromosome 3; *LpWRKY70* to *LpWRKY75* (group III), *LpWRKY76* to *LpWRKY79* (group III) and *LpWRKY80* to *LpWRKY81* (group IIb) were located on chromosome 5.

**Figure 5:**
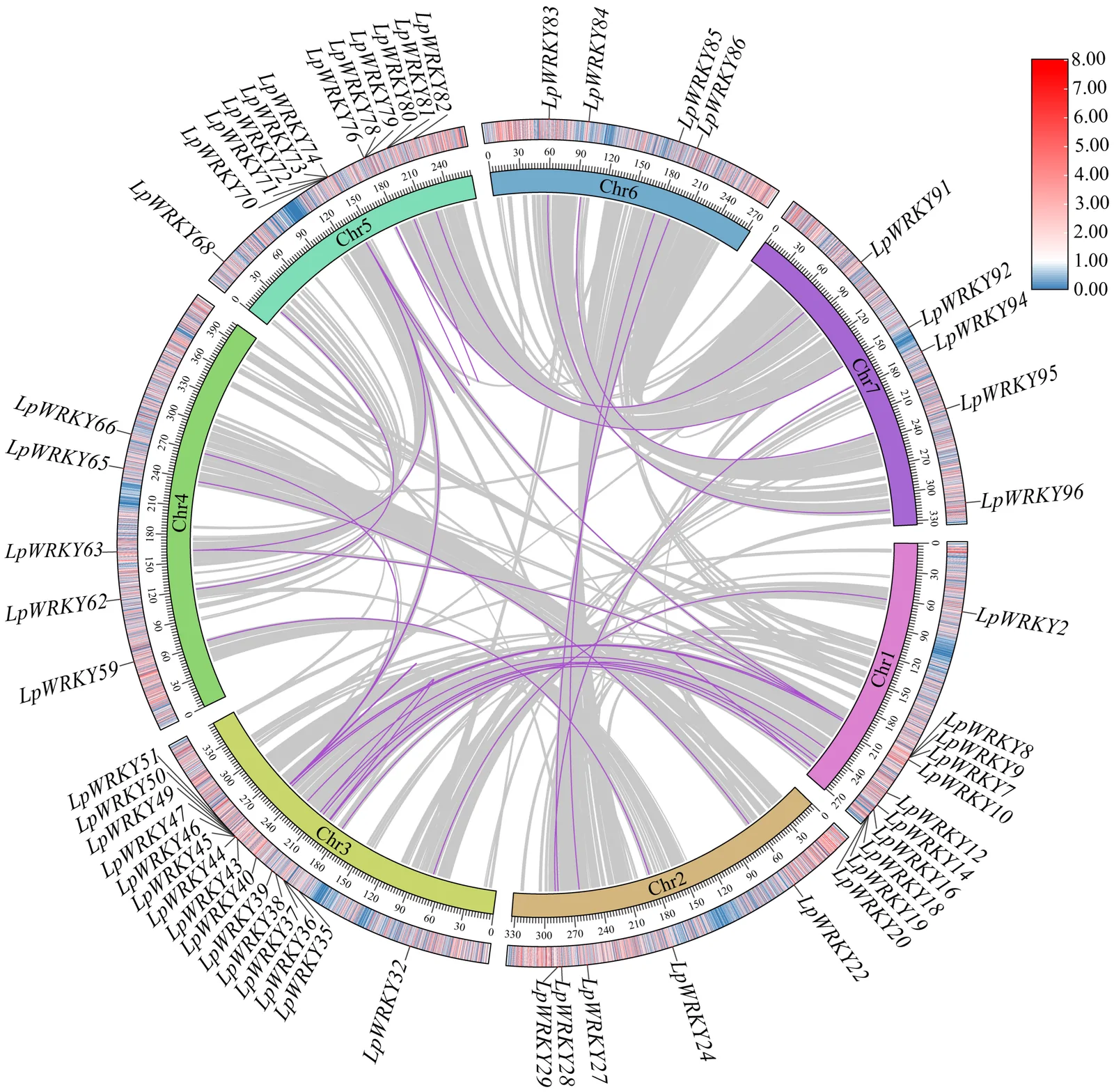
Collinearity analysis of *WRKY* gene family members in perennial ryegrass by TBtools-II (v2.326) with MCScanX, illustrating genomic duplication events among homologs. Duplicated gene pairs were connected with purple lines.

To investigate the expansion of the *WRKY* gene family in perennial ryegrass, TBtools-II (v2.326) with MCScanX was employed to analyze gene duplication patterns including tandem duplications and segmental duplications. Among the 97 genes, 12 tandem duplications were detected, distributed on chromosomes 1, 3 and 5 (Figure 5; Table S4). Notably, ten pairs of tandem duplicated genes belonged to group III, while two tandem duplicated gene pairs were assigned to group IIa and IIe respectively. Apart from tandem duplication events, 27 segmental duplications were identified across different perennial ryegrass chromosomes, further confirming that the *LpWRKY* gene family is highly conserved. These events suggested that some *LpWRKY* genes may have originated through gene replication events, which might contribute to the evolutionary expansion and functional differentiation of the *LpWRKY* gene family.

The nonsynonymous substitution rate (Ka), synonymous substitution rate (Ks) and Ka/Ks ratio for the duplicated genes were calculated to evaluate the selective pressure acting on the *LpWRKY* family during evolution. The Ka/Ks values of all *LpWRKY* duplication ranged from 0.18 to 0.63 (Table S4). The results revealed that *LpWRKY* gene family was affected by purifying selection during evolution, considering Ka/Ks ratios less than 1.

**Figure 6:**
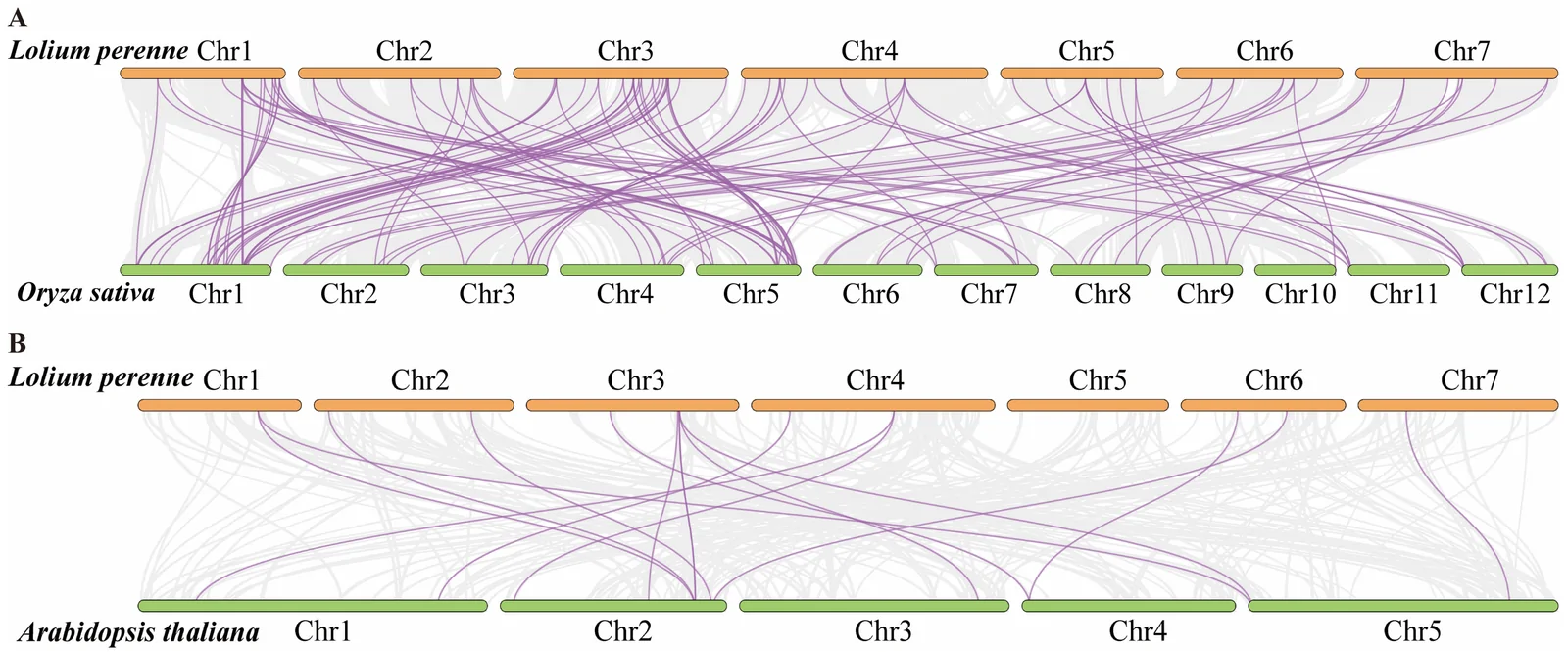
Interspecifc collinearity analysis of WRKY gene family by TBtools-II (v2.326) with MCScanX. (**A**) Collinearity between perennial ryegrass and *Oryza sativa*. (**B**) Collinearity between perennial ryegrass and *Arabidopsis thaliana*. Purple lines indicate orthologous gene pairs.

To further investigate the evolutionary conservation and interspecific homology of the perennial ryegrass *WRKY* family, the syntenic maps of perennial ryegrass in association with one monocot (rice) and one dicot (*Arabidopsis*) were constructed respectively. Among the 97 *LpWRKY* genes and 102 *OsWRKY* genes, a total of 117 syntenic gene pairs were identified across all seven perennial ryegrass chromosomes (Figure 6A). The highest number of syntenic gene pairs (32 pairs) located on perennial ryegrass chromosome 3 involving 19 *LpWRKY* genes and 29 *OsWRKY* genes (Table S5). Six *LpWRKY* genes exhibited extensive syntenic relationships with rice *WRKY* genes, forming at least three collinear pairs each: *LpWRKY7*(3 pairs), *LpWRKY18*(3 pairs), *LpWRKY70*(3 pairs), *LpWRKY86*(3 pairs), *LpWRKY37*(4 pairs) and *LpWRKY66*(5 pairs). And interestingly, of these six *LpWRKY* genes, three were in group III, including *LpWRKY7*, *LpWRKY70* and *LpWRKY66*. This may indicate a high conservation of genomic relationships among group III *WRKY* genes within Poaceae plants. In contrast, the smaller number of collinear gene pairs was found between 97 genes in perennial ryegrass and 72 genes in *Arabidopsis* (Figure 6B). 14 *LpWRKY* genes formed 16 collinearity pairs with 12 *WRKY* genes from *Arabidopsis* (Table S5). The number of collinear *LpWRKY* genes was significantly higher in rice than in *Arabidopsis*, reflecting that many *WRKY* genes arose before the divergence of the rice and perennial ryegrass lineages.

### 2.6. Cis-Acting Elements in the Promoters of LpWRKY Genes

**Figure 7:**
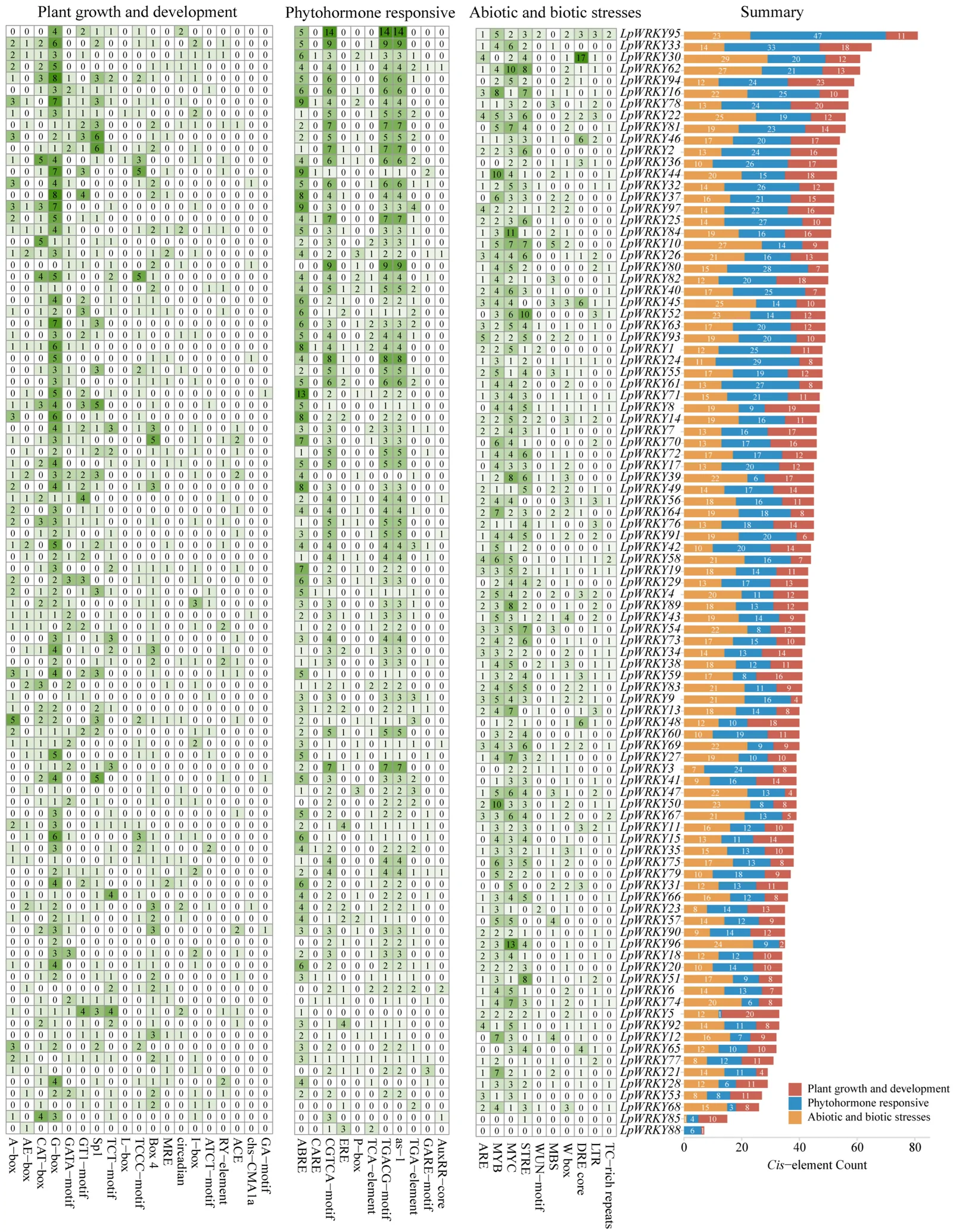
Promoter *cis*-acting element distribution analysis of *WRKY* gene family members from perennial ryegrass. Elements are grouped into three functional categories, plant growth and development-related elements, phytohormone-responsive elements, and stress-responsive elements. The intensity of the color refects the abundance of each element within the corresponding promoter.

To explore the potential regulatory mechanisms and biological functions of *LpWRKY* genes, the putative *cis*-acting elements in these promoters were predicted using PlantCARE website (https://bioinformatics.psb.ugent.be/webtools/plantcare/html/, accessed on 31 August 2026) and visualized via TBtools-II (v2.326). The 2000 bp upstream sequence of each *LpWRKY* gene start codon (ATG) was selected as the promoter sequence. In addition to core promoter elements such as the CAAT-box and TATA-box, which are essential for transcription initiation and enhancers, various *cis*-acting elements were found to be concentrated in the promoter region of *LpWRKYs*, associated with responses to biotic and abiotic stresses. A total of 41 *cis*-acting elements were predicted and classified into three functional categories based on their potential regulatory activity: plant growth and development, phytohormonal responses, and biotic and abiotic stress responses (Figure 7).

Plant growth and development *cis*-acting elements, including G-box (Light responsiveness), GT1-motif (Light responsiveness) and CAT-box (meristem expression), are widely distributed in *LpWRKY* promoter regions (Figure 7). Phytohormone-responsive *cis*-acting elements contained abscisic acid (ABA) responsive element (ABRE), gibberellin (GA)-responsive element (CARE), methyl jasmonate (MeJA)-responsive element (CGTCA-motif and TGACG motif), growth hormone-responsive element (AuxRR-core and TGAelement), gibberellin-responsive elements (GARE-motif and P-box), and salicylic acid-responsive element (TCA-element and as1), covering multiple hormone signaling pathways. Among these hormone-related elements, MeJA-responsive elements and ABA-responsive elements accounted for the largest portion: 96% (93/97) of *LpWRKYs* harbored MeJA-responsive elements and 91% (89 /97) of the genes contained the ABRE motif. Furthermore, abundant stress-responsive *cis*-acting elements were detected, including ARE (anaerobic induction), LTR (low-temperature responsiveness), STRE (stress responsive elements), TC-rich repeat (defense and stress responsiveness), MBS (an MYB transcription factor binding site involved in drought inducibility) and WUN-motif (wound-responsive element). Collectively, these predicted *cis*-acting element features imply a potential association of *LpWRKY* genes with diverse biotic and abiotic stress-response pathways in perennial ryegrass.

### 2.7. The Protein–Protein Interaction Network of LpWRKY

**Figure 8:**
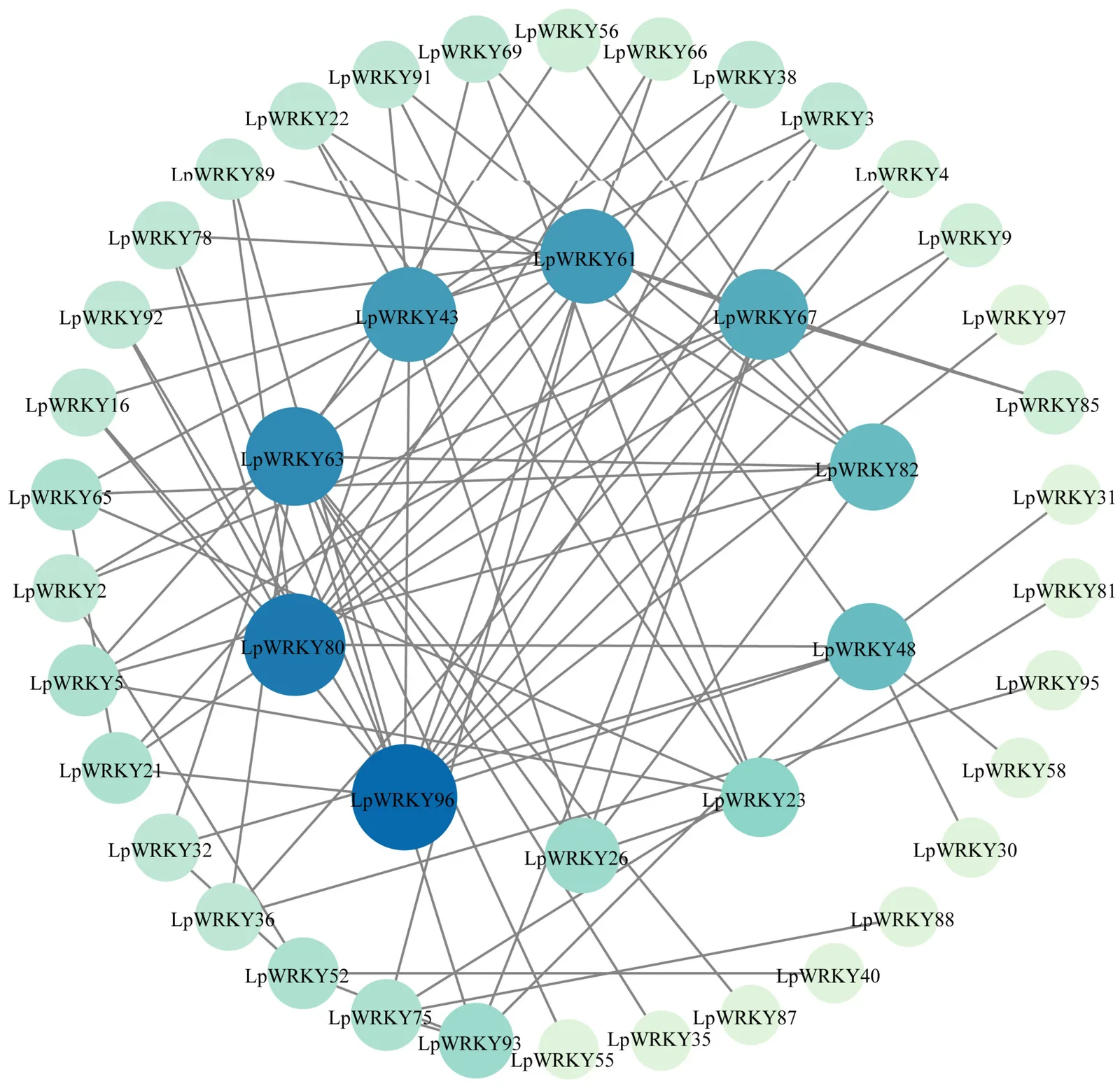
Analysis of the protein interaction network of LpWRKYs. All data were obtained from the STRING database.

To analyze the synergistic effects of LpWRKY transcription factors in the regulatory process, a protein-protein interaction (PPI) network was constructed using TBtools-II (v2.326) through the STRING database, The predicted network analysis showed that LpWRKY96, LpWRKY80 and LpWRKY63 were the top three hub proteins with the most interaction partners, harboring 13, 12, and 11 interacting proteins, respectively (Figure 8). LpWRKY96 exhibited the highest interaction frequency, implying a potential role as a putative core hub that may modulate multiple downstream signaling pathways under stress, which remains to be experimentally verified.

### 2.8. Expression Patterns of LpWRKYs in Roots and Leaves Under Heat Stress

**Figure 9:**
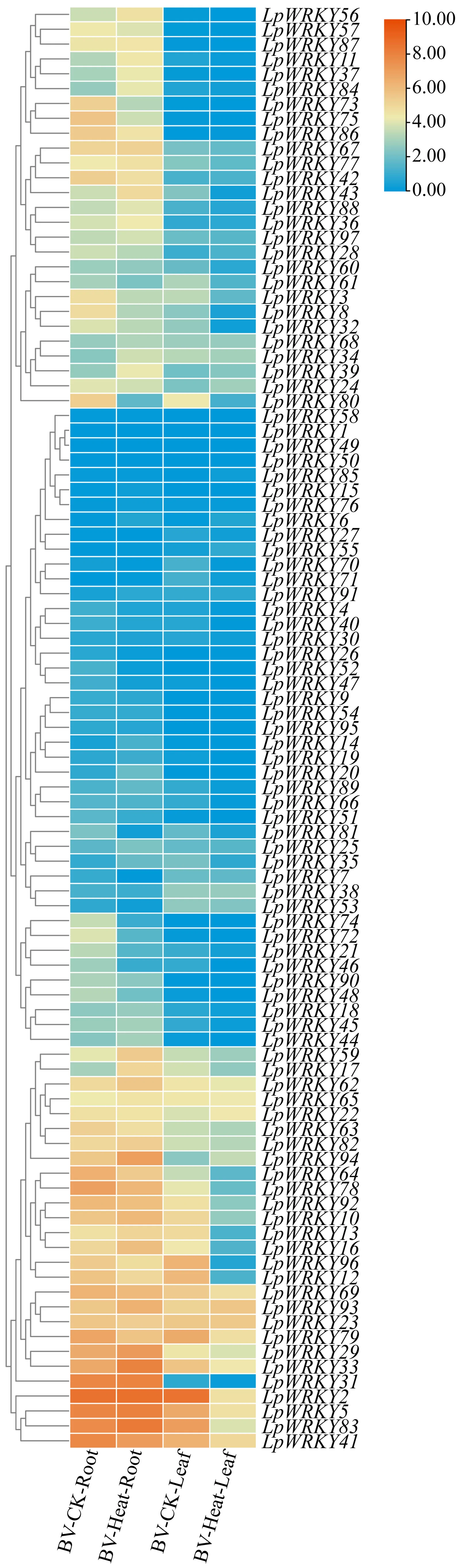
Expression heatmap of all *LpWRKY* genes in different tissues of perennial ryegrass under heat stress. The heatmap was generated based on TPM values by TBtools-II (v2.326).

**Figure 10:**
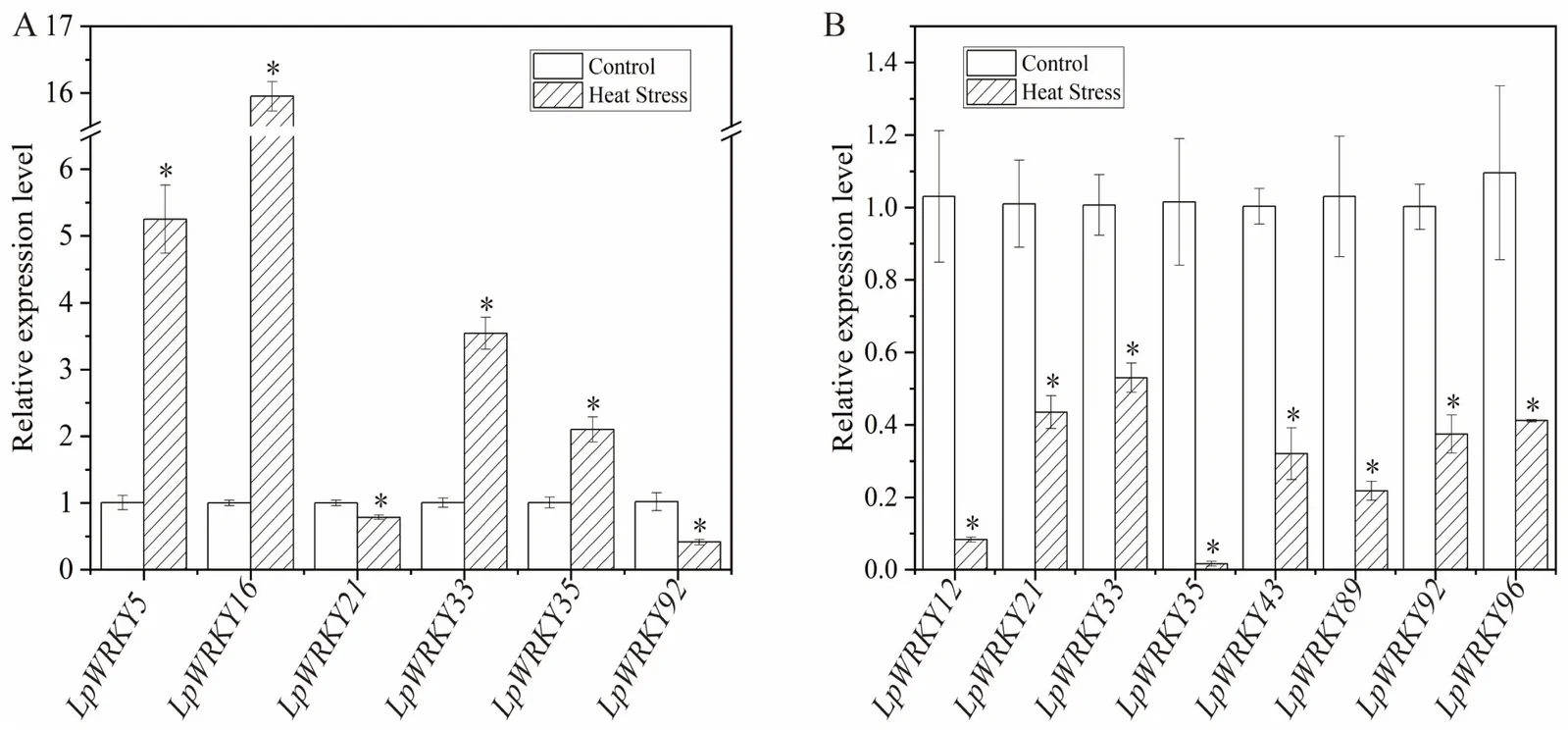
qRT-PCR expression level of *LpWRKYs* in roots (**A**) and leaves (**B**) of perennial ryegrass under heat stress. An endogenous reference gene *LpeIF4A* (GenBank: GO924770.1) was uesd for data normalization. Error bars represent the standard error of the means for three biological replicates. Asterisks indicate significant differences between control and heat stress determined by Student’s *t*-test (* *p* < 0.05).

Since the heat tolerance is of great significance for perennial ryegrass, we further focused on the analysis of gene expression patterns under heat stress. We analyzed the RNA-Seq data in perennial ryegrass cultivar ‘BV’ under normal and high temperature conditions [26]. Based on TPM values, the heatmap illustrated the expression patterns of *LpWRKY* genes in roots and leaves in response to heat stress. In roots, 14 *LpWRKY* genes exhibited significant down-regulation and 11 showed significant up-regulation (Figure 9). In leaves, 31 *LpWRKY* genes were markedly down-regulated and only 1 gene was up-regulated under heat stress (Figure 9). Among these *LpWRKY* genes identified by RNA-Seq analysis in perennial ryegrass, we randomly selected *LpWRKY* genes from different groups to futher validate their expression profiles in response to heat stress using qRT-PCR (Figure 10). The results showed that the expression levels of *LpWRKY5*, *LpWRKY16*, *LpWRKY33* and *LpWRKY35* were significantly up-regulated and *LpWRKY21* and *LpWRKY92* were significantly down-regulated in roots after applying heat stress to for 24 h (Figure 10A). The transcript levels of *LpWRKY* genes *LpWRKY12*, *LpWRKY21*, *LpWRKY33*, *LpWRKY35*, *LpWRKY43*, *LpWRKY89*, *LpWRKY92* and *LpWRKY96* displayed significant down-regulation in leaves under heat stress (Figure 10B). The qRT-PCR findings were in accordance with the expression profiles obtained from the RNA-seq data. The expression changes of these genes in roots and leaves may imply their involvement in the heat-stress response of perennial ryegrass. Interestingly, the expression levels of *LpWRKY33* and *LpWRKY35* exhibited opposite responses in roots and leaves under heat stress, suggested substantial tissue specificity in the WRKY-mediated heat-stress response.

## 3. Discussion

As far as is known, WRKY is one of the largest families of regulatory proteins in plants, participating in a variety of physiological processes ranging from developmental programs to stress and defense responses [2,27,28]. Due to the agronomic and ecological importance of perennial ryegrass as a cool-season forage and turf species extensively cultivated in various temperate regions, a comprehensive genome-wide characterization of its *WRKY* family was accomplished, providing the first systematic overview of their phylogenetic relationships, structural features, evolutionary dynamics, and expression responses to heat stress.

### 3.1. Identification and Characterization of LpWRKY Members

In this study, a total of 97 *LpWRKY* genes were finally identified from the latest perennial ryegrass genome database, which were named as *LpWRKY1* to *LpWRKY97* based on their chromosomal positions. The number of *WRKY* gene in perennial ryegrass is greater than that in *Arabidopsis* but fewer than that in rice. There was no significant correlation between the number of *WRKY* genes and genome size, for example, *Arabidopsis* genome size 135 Mb (75 *WRKY* genes), rice genome size 386 Mb (101 *WRKY* genes) [29], *Cenchrus purpureus* genome size 1.97 Gb (176 *WRKY* genes) [10,30], *Medicago sativa* genome size 816 Mb (91 *WRKY* genes) [16,31] and *Lolium perenne* genome size 2.26 Gb (97 *WRKY* genes) [32], indicating that the evolutionary development of the WRKY family varies among different plant species. The quantity of perennial ryegrass WRKY family members might be linked to its evolutionary process and the pattern of family expansion, while the quality of genome assembly also served as a significant factor influencing the gene count.

Based on the canonical classification system established in rice and *Arabidopsis* [3,4,25], the 97 LpWRKY proteins were divided into three major groups (I, II and III), with group II further subdivided into five subgroups (IIa–IIe). The proportions of WRKY varied across each group. Notably, group II contained the largest number of *LpWRKYs* (48 genes), while group III had a slightly lower number (37 genes). The higher proportion of group II *WRKY* genes was consistent with observations in rice (60/102 *OsWRKYs*) and elephant grass (84/176 *CpWRKYs*) [10], suggesting that this group has undergone extensive expansion, which is likely driven by lineage-specific duplications to adapt to diverse regulatory requirements.

### 3.2. Evolutionary Expansion and Purifying Selection

Gene duplication, including both tandem and segmental events, are thought to contribute to gene-family expansion and functional innovation. In perennial ryegrass, we identified 12 tandem duplication events and 27 segmental duplication events, collectively accounting for a substantial proportion of the entire *LpWRKY* family. Notably, tandem duplications were predominantly confined to chromosomes 3 and 5, forming gene clusters that may have arisen from recent duplication events. The Ka/Ks ratios for all duplicated gene pairs were significantly less than 1, ranging from 0.18 to 0.63. It is indicated that strong purifying selection had acted upon the *LpWRKY* family throughout evolution. The prevalence of purifying selection is consistent with reports on *WRKY* families in maize and rice [33], supporting the view that these TFs perform essential regulatory functions that are largely constrained during evolution.

In addition, we analyzed the collinear relationships between perennial ryegrass and rice and *Arabidopsis*. In these two comparative analyses, the perennial ryegrass *WRKY* family was found to have stronger collinearity with rice and weaker collinearity with *Arabidopsis* (Figure 6). These results indicated that perennial ryegrass shared more genomic linkages with monocotyledonous plants compared to dicotyledonous plants. Notably, most *WRKY* genes from perennial ryegrass could be mapped to duplicated segments in its congeneric species rice. This indicates that the *LpWRKY* gene family is relatively evolutionarily conserved, whereas whether their functional and structural features are highly similar to those of other Poaceae members remains to be explored. Several *LpWRKY* genes, particularly those belonging to group III (e.g., *LpWRKY76*, *LpWRKY66* and *LpWRKY70*), exhibited multiple collinear relationships with *OsWRKYs*. This collinear pattern raises the hypothesis that these genes may play conserved roles in Poaceae-specific physiological processes such as stress responses or development, although this prediction requires experimental validation.

### 3.3. Structural Conservation and Localization of the LpWRKY Family

The highly conserved WRKY domain, characterized by the WRKYGQK heptapeptide at the N-terminus and a zinc-finger motif at the C-terminus, was present in all identified LpWRKY proteins. Group I contained two WRKY conserved structural domains and the C-X4-5C-X22-23-HXH zinc-finger motif, group II contained one WRKY conserved structural domain and one Cx4-5Cx22-23HxH zinc-finger motif, and group III contained one WRKY conserved structural domain and the Cx7Cx23HxC zinc-finger motifs. In addition, amino acid variants of WRKY domains were detected in several group II and III members, including WRKYGKK, WRKYGEK, WRKYGXK and WRIYGQK. Similar substitutions have been documented in rice, *Ricinus communis*, elephant grass and *P. tenuifolia* [10,34,35,36]. Although the functional alternations of these LpWRKY variants remain to be experimentally verified, previous studies have shown that the WRKY TFs with WRKYGKK mutation abolished W-box binding affinity, as demonstrated in GmWRKY6, GmWRKY21 and GmWRP1 [37,38]. WRKY TFs regulate gene expression by binding specifically to the W-box in the promoter region of the target gene. Hence, it is plausible that a subset of LpWRKY proteins may have lost canonical DNA-binding activity and acquired novel regulatory mechanisms, potentially diversifying their roles within the regulatory network.

WRKY proteins are typically localized in the cell nucleus, where they regulate the expression of target genes in plants. Based on the predicted subcellular localization results of the 97 LpWRKYs, we observed that they were localized in the nucleus, peroxisome, chloroplast, cytoplasm and mitochondrion (Table 1). The majority of LpWRKY proteins (84/97, 86.6%) were localized within the nucleus, which is consistent with their roles as transcription factors. The 13 LpWRKY proteins displaying diverse subcellular localization patterns may have multiple functions and mechanisms. Notably, WRKY TFs located at other subcellular sites have been associated with novel functions [37,39].

### 3.4. Promoter Architecture and Putative Regulatory Roles

*Cis*-acting elements are specific binding sites involved in initiating and regulating the transcription of a gene. The promoter regions of *LpWRKY* genes harbored a diverse array of *cis*-acting elements, reflecting their multiple functions in the regulatory network. Besides the core elements CAAT-box and TATA-box, we predicted numerous elements associated with development, phytohormone signaling, and stress response. MeJA- and ABA-responsive elements were more widely distributed, along with light-responsive G-box and stress-related elements such as LTR and STRE. The overwhelming prevalence of MeJA (found in 96% of *LpWRKYs*) and ABA-responsive elements (found in 91% of *LpWRKYs*) is noteworthy, as both hormones are pivotal in orchestrating plant responses to abiotic stresses, including heat and drought. This *cis*-regulatory landscape suggests that *LpWRKYs* may possess the potential to enable rapid and coordinated transcriptional reprogramming in response to environmental perturbation. However, the absence of canonical HSEs (heat shock element) in many promoters, which are known to be activated by heat shock transcription factors, indicates that their responsiveness to heat stress could be mediated indirectly through other transcription factors or signaling cascades.

### 3.5. Heat-Responsive LpWRKYs and Their Potential as Thermotolerance Regulators

Heat stress is a major constraint for cool-season grasses, such as perennial ryegrass. Under heat stress, perennial ryegrass exhibits slowed growth, reduced forage yield and deteriorated turf quality. However, the molecular mechanisms underlying thermotolerance remain largely unexplored. *WRKY* genes are widely involved in regulating stress responses across various plant species, and considered as prominent candidate genes for genetic engineering to improve abiotic stress tolerance. Thus, gene expression profiles were analyzed to obtain heat stress-responsive LpWRKY TFs and elucidate their potential functions in perennial ryegrass. Through transcriptomic and qRT-PCR analysis, ten *LpWRKY* genes were confirmed to respond to heat stress treatments in roots or/and leaves. It indicates that these genes may play potential roles in perennial ryegrass resistance to heat stress and further functional exploration is necessary. These heat-responsive WRKY TFs may be activated or suppressed to regulate the activation of downstream protective mechanisms. Many previously characterized WRKYs in other plants acted as positive regulators of heat tolerance, such as AtWRKY25/26/33/39 in *Arabidopsis*, TaWRKY33/70 in wheat, SlWRKY3 in tomato and StWRKY75 in potato [17,18,19,20,21,22,24]. Whereas, repression of *WRKY* genes has also been linked to heat adaptation; for instance, AtWRKY48 negatively regulates acclimation to combined high light and heat stress [40], *LpWRKY69* and *LpWRKY70* (referred to as *LpWRKY92* and *LpWRKY10* in the present study) were proposed to negatively regulate heat-induced leaf senescence, conferring heat tolerance in perennial ryegrass [41].

Furthermore, the expression profiles demonstrated that most of *LpWRKY* genes were down-regulated after heat stress treatment for 24 h, while fewer *LpWRKY* genes were up-regulated. Noticeably, the expression of the *LpWRKY33* and *LpWRKY35* gene exhibited differential response to heat stress between the leaves (down-regulation) and roots (up-regulation) in perennial ryegrass. As the main photosynthetic organ, leaves are directly exposed to high-temperature stress, whereas roots perceive underground thermal signals and mediate systemic stress communication across the whole plant. The tissue-specific transcriptional profiles of these two genes suggest they may execute diverse and tissue-specific functions as components of complex regulatory networks responsing to heat stress, instead of serving as general heat-responsive regulators. However, the underlying mechanisms of these *LpWRKY* gene functions still remain to be further elucidated. The distinct expression patterns in roots and leaves provide valuable candidates for further exploring the regulatory networks underlying heat-stress responses in perennial ryegrass.

Among these heat stress-related genes, *LpWRKY96* emerges as a putative hub gene, not only due to its significant down-regulation but also because it exhibited the highest level of interaction with other LpWRKY TFs based on predicted protein-protein interaction network. Notably, in the present study, *LpWRKY96* was positioned at the center of an interaction network formed with four other down-regulated *LpWRKYs* in leaves, namely *LpWRKY21*, *LpWRKY43*, *LpWRKY89*, and *LpWRKY92*. This suggests that LpWRKY96 might hypothetically function as a central coordinator within the transcriptional regulatory network under high temperature conditions, possibly alleviate repression of downstream effectors. Whether this module directly affects heat tolerance through targeting antioxidant-related genes, heat shock proteins, or hormone metabolism pathways remains to be determined via future functional studies, such as knockout or overexpression experiments in perennial ryegrass.

## 4. Materials and Methods

### 4.1. Identification of the WRKY Gene Family

The genome and GFF files of perennial ryegrass were downloaded from NCBI (https://www.ncbi.nlm.nih.gov/datasets/genome/GCF_019359855.2/, accessed on 25 August 2026), and the protein file was obtained using TBtools-II (v2.326). The Hidden Markov Model (HMM) file for WRKY (PF03106) was downloaded from https://www.ebi.ac.uk/interpro/entry/pfam/PF03106/ (accessed on 25 August 2026). WRKY genes in perennial ryegrass were identified by a combination of HMMER and BLAST homology searches. HMMER searches were performed with an E-value threshold of ≤1 × 10^−5^. For BLAST searches, WRKY protein sequences from *Arabidopsis thaliana* and *Oryza sativa* were used as query sequences, with a bit score ≥100 and E-value <10^−5^. Redundant sequences from the two search results were removed. The identified genes were then verified via CD-Search (https://www.ncbi.nlm.nih.gov/Structure/bwrpsb/bwrpsb.cgi, accessed on 25 August 2026) to confirm their conserved structural domains (E-value < 0.01). Physicochemical properties of the confirmed proteins were predicted using the ProtParam tool (ExPASy; https://web.expasy.org/protparam/, accessed on 25 August 2026) [42]. The chromosomal localization was generated by TBtools-II (v2.326). WoLF PSORT (WoLF PSORT: Protein Subcellular Localization Prediction, https://wolfpsort.hgc.jp/, accessed on 28 August 2026) was used to predict the subcellular localization of LpWRKYs.

Based on the annotation file, we extracted gene IDs and their corresponding alternative splicing (AS) events. The number of transcript isoforms per gene was computed in R v4.5.1 using the “GenomicFeatures” package. Genes with two or more transcript isoforms were considered genes with potential alternative-splicing events. When multiple splice variants were annotated for a single locus, the representative isoform was chosen as the longest protein sequence containing the complete WRKY conserved domain.

All gene structures, chromosome coordinates, AS events, and other annotation information used in the subsequent analyses were obtained from the perennial ryegrass genome annotation of NCBI (Kyuss_2.0).

### 4.2. Phylogenetic Analysis

The amino acid sequence of *Arabidopsis* and rice WRKY proteins were accessed from the plantTFDB online database (https://planttfdb.gao-lab.org, accessed on 28 August 2026). The multiple sequence alignment was performed using ClustalW 2.1, Gaps and poorly-aligned regions were trimmed by trimAl v1.4.1. The maximum-likelihood phylogenetic tree was inferred using FastTree v2.1.10 under the JTT (Jones-Taylor-Thornton, 1992) amino-acid substitution model with the CAT approximation for site-specific rate variation. The reliability of internal branches was evaluated using SH-like local support values with 1000 site resamples [43].

### 4.3. Conserved Motifs and Gene Structure Analysis

The MEME (MutipleEm for Motif Elicitation) analysis was performed using the online tool at https://meme-suite.org/meme/tools/meme (accessed on 29 August 2026), and the conserved domain search was conducted using https://www.ncbi.nlm.nih.gov/Structure/bwrpsb/bwrpsb.cgi (accessed on 29 August 2026). The maximum number of motifs was set to 10, and the motif length was set from 6 to 200 amino acids. Other parameters were kept as default settings. Based on the complete genome annotation file, the TBtools-II (v2.326) with Gene Structure View (Advanced) was utilized to conduct the examination of the exon-intron composition of *LpWRKY* genes with default parameters. TBtools-II (v2.326) was used for clustering and visualization of the results, which generated the phylogenetic tree, conserved motifs, gene structure, and conserved domain analysis of *LpWRKY* genes in perennial ryegrass.

### 4.4. Chromosomal Location, Gene Duplication and Synteny Analysis

The chromosomal locations of the *LpWRKY*s were extracted from the GFF annotation files of perennial ryegrass, and visualized using the Gene Location Visualize from GTF/GFF module implemented in TBtools-II (v2.326).

TBtools-II (v2.326) with the built-in One-Step MCScanX module was used to analyze the collinear blocks and duplication events of *LpWRKYs* with *OsWRKYs* and *AtWRKYs*. TBtools-II (v2.326) was used to calculate the ratio of nonsynonymous substitution rate (Ka) to synonymous substitution rate (Ks) (Ka/Ks). Tandem duplicated gene pairs were defined as homologous genes located with no more than one intervening gene. Genes residing within collinear syntenic blocks were regarded as segmental duplication pairs [44].

### 4.5. Promoter Analysis

The 2000 bp sequences upstream of the start codon (ATG) of *LpWRKY* genes were extracted as the promoter region due to the lack of high-quality public transcription start site (TSS) annotation in perennial ryegrass genome. *Cis*-acting elements were predicted using PlantCARE website (https://bioinformatics.psb.ugent.be/webtools/plantcare/html/, accessed on 31 August 2026) [45], and visualization analysis was performed by TBtools-II (v2.326).

### 4.6. Protein-Protein Interaction Prediction

The interactions among LpWRKY proteins in perennial ryegrass were predicted using TBtools-II (v2.326) software and the String database (https://string-db.org/, accessed on 3 September 2026). Full-length sequences of the 97 LpWRKY proteins identified in this study served as the primary input. *Oryza sativa Japonica* was selected as the reference organism. Network display settings were configured to show query protein names and hide disconnected nodes. All other parameters were kept at default values.

### 4.7. RNA-Seq Analysis of LpWRKYs Under Heat Stress

Roots and leaves samples of perennial ryegrass cultivar ‘BV’ were harvested for RNA sequencing in our previous study, subjected to 24 h of heat stress in the growth chamber at 38/30 °C (day/night) or 25/20 °C for the control [26]. Three biological repeats were taken for each group. The publicly available RNA-seq data from the NCBI SRA database (https://www.ncbi.nlm.nih.gov/sra, accessed on 3 September 2026; SRA accession number: PRJNA1278810) were downloaded and analyzed. Sequencing reads were mapped to the reference genome (NCBI_GCF_019359855.1) using HISAT2. Expression levels of the *LpWRKY* genes were quantified using the transcript per million (TPM) method, and visualized in a heatmap using TBtools-II (v2.326). Differentially expressed genes (DEGs) were identified using DESeq2 (v1.24) software with the selection criteria set to |Log2FC| ≥1 and FDR <0.01.

### 4.8. qRT-PCR Analysis of LpWRKYs Under Heat Stress

For qRT-PCR validation, independent plant materials were sampled, which were independent from the samples used for RNA-seq analysis. Perennial ryegrass cultivar ‘BV’ seedlings were grown at 25/20 °C (day/night) for the control group or 38/30 °C for the heat-stress treatment for 24 h. Roots and leaves were collected and immediately frozen in liquid nitrogen. Total RNA was extracted using Trizol reagent (TianGen, Nanjing, China). The extracted RNA was used for cDNA synthesis with the ReverTran Ace^®^ qPCR RT kit (Vazyme, Nanjing, China). qRT-PCR was performed in a 20 μL reaction volume using SYBR^®^ Green Real-time PCR Master Mix (Vazyme, Nanjing, China) on the QuantStudio 5 system (Thermo Fisher, Waltham, MA, USA). The primers were listed in Table S6. *LpeIF4A* was used as an internal control, which was verified as the most suitable reference gene under heat stress [46]. Three independent biological replicates were included, with each replicate derived from a single individual plant.

Statistical analyses were performed using SPSS 27.0 software (IBM Corp., Armonk, NY, USA); all values were expressed as mean ± SE (*n* = three independent biological replicates). Relative expression levels were calculated using the 2^−∆∆Ct^ method [47], with an endogenous reference gene used for normalization. Statistical significance was determined by Student’s *t*-test (*p* < 0.05).

## 5. Conclusions

In this study, we identified 97 *LpWRKY* genes in perennial ryegrass, which were classified into three main groups. Members within the same group exhibited highly similar motif compositions and exon-intron structures. These genes were unevenly distributed across the chromosomes. Moreover, perennial ryegrass shared more genomic linkages with monocotyledonous plants than with dicotyledonous plants. The promoters of LpWRKYs contained a variety of *cis*-acting elements associated with stress responses, hormone signaling, and growth regulation. The expression patterns of *LpWRKYs* in roots and leaves suggested that they may play potential roles in heat stress responses. Notably, *LpWRKY33* and *LpWRKY35* exhibited tissue-specific transcriptional patterns under heat stress, implying potential participation in complex regulatory networks. LpWRKY96 may serve as a potential central coordinator in the heat response signaling network. This study systematically characterizes the LpWRKY gene family and offers valuable candidate genes for future functional verification and molecular improvement of heat tolerance in perennial ryegrass.

## Figures and Tables

**Table 1:** Complete list of *LpWRKY* genes identified in perennial ryegrass.

Gene Name	Gene ID	Chr. No.	Start Position (bp)	End Position (bp)	CDS Length (bp)	Protein Length (aa)	Subcellular Locaion	Group
*LpWRKY1*	LOC127326027	1	10363444	10366913	927	309	Chloroplast	III
*LpWRKY2*	LOC127295137	1	61320439	61321321	606	202	Nucleus	IIc
*LpWRKY3*	LOC127297109	1	72286797	72288292	945	315	Nucleus	III
*LpWRKY4*	LOC127294403	1	80502033	80506852	1170	390	Nucleus	III
*LpWRKY5*	LOC127303171	1	81044446	81046325	1419	473	Nucleus	I
*LpWRKY6*	LOC127312231	1	168711537	168713095	1008	336	Nucleus	IIe
*LpWRKY7*	LOC127319033	1	200221723	200225310	870	290	Nucleus	III
*LpWRKY8*	LOC127319059	1	200238897	200240051	924	308	Nucleus	III
*LpWRKY9*	LOC127311896	1	200390460	200391541	855	285	Nucleus	III
*LpWRKY10*	LOC127319348	1	202111196	202113891	1527	509	Nucleus	I
*LpWRKY11*	LOC127326309	1	231900521	231903528	651	217	Nucleus	IIc
*LpWRKY12*	LOC127325184	1	237351294	237352753	699	233	Cytoplasm	IIc
*LpWRKY13*	LOC127325148	1	237592098	237593441	687	229	Cytoplasm	IIc
*LpWRKY14*	LOC127327623	1	251088514	251091610	1236	412	Nucleus	IIc
*LpWRKY15*	LOC127327656	1	253529947	253534124	1827	609	Chloroplast	IIb
*LpWRKY16*	LOC127327709	1	255753347	255754350	744	248	Mitochondrion	III
*LpWRKY17*	LOC127327711	1	255905723	255906673	753	251	Mitochondrion	III
*LpWRKY18*	LOC127327789	1	262310650	262312820	930	310	Nucleus	IIc
*LpWRKY19*	LOC127327739	1	262553843	262555202	894	298	Nucleus	IIe
*LpWRKY20*	LOC127327755	1	262606365	262607443	840	280	Nucleus	IIe
*LpWRKY21*	LOC127331497	2	24220211	24224743	1041	347	Nucleus	III
*LpWRKY22*	LOC127332793	2	63349943	63353428	1278	426	Nucleus	I
*LpWRKY23*	LOC127332871	2	68275419	68282224	1833	611	Nucleus	I
*LpWRKY24*	LOC127334106	2	184958537	184961459	846	282	Nucleus	IIc
*LpWRKY25*	LOC127334322	2	196860615	196864836	2853	951	Cytoplasm	III
*LpWRKY26*	LOC127335225	2	233482960	233486620	2259	753	Nucleus	I
*LpWRKY27*	LOC127335803	2	262431857	262437395	702	234	Nucleus	IIc
*LpWRKY28*	LOC127336379	2	285312980	285317437	1467	489	Nucleus	IIe
*LpWRKY29*	LOC127336323	2	288524229	288525646	1047	349	Peroxisome	IId
*LpWRKY30*	LOC127341622	3	67204032	67209128	774	258	Chloroplast	IIc
*LpWRKY31*	LOC127341560	3	69787531	69789968	1506	502	Nucleus	IIb
*LpWRKY32*	LOC127341558	3	70025868	70028010	612	204	Nucleus	IIc
*LpWRKY33*	LOC127342204	3	118143411	118146281	1710	570	Nucleus	IIb
*LpWRKY34*	LOC127342597	3	139380974	139386769	1608	536	Nucleus	IIb
*LpWRKY35*	LOC127342754	3	180857383	180858495	729	243	Nucleus	IIc
*LpWRKY36*	LOC127343013	3	196998265	197000818	969	323	Nucleus	IIe
*LpWRKY37*	LOC127343024	3	197805656	197807745	1032	344	Nucleus	IIc
*LpWRKY38*	LOC127343169	3	206525621	206527023	1041	347	Nucleus	III
*LpWRKY39*	LOC127343227	3	210500736	210503431	1095	365	Nucleus	IIc
*LpWRKY40*	LOC127338566	3	228935263	228937664	699	233	Chloroplast	IIc
*LpWRKY41*	LOC127343866	3	239964165	239965591	933	311	Nucleus	IIe
*LpWRKY42*	LOC127344604	3	246721278	246723230	912	304	Nucleus	IIe
*LpWRKY43*	LOC127344745	3	252297172	252299740	1686	562	Nucleus	I
*LpWRKY44*	LOC127338743	3	254753357	254754673	813	271	Nucleus	III
*LpWRKY45*	LOC127338744	3	254859441	254860809	819	273	Nucleus	III
*LpWRKY46*	LOC127344792	3	254973260	254975408	930	310	Nucleus	III
*LpWRKY47*	LOC127344793	3	255029191	255031238	966	322	Nucleus	III
*LpWRKY48*	LOC127338745	3	255155455	255156757	813	271	Nucleus	III
*LpWRKY49*	LOC127340119	3	255329272	255332250	1143	381	Nucleus	III
*LpWRKY50*	LOC127340120	3	255526808	255528813	984	328	Nucleus	III
*LpWRKY51*	LOC127338746	3	255585417	255591831	798	266	Nucleus	III
*LpWRKY52*	LOC127338747	3	255642918	255648066	783	261	Nucleus	III
*LpWRKY53*	LOC127344493	3	274203727	274208480	930	310	Nucleus	IIa
*LpWRKY54*	LOC127346233	3	350985245	350988017	1101	367	Nucleus	IIa
*LpWRKY55*	LOC127349404	4	5848867	5851728	1143	381	Nucleus	IIe
*LpWRKY56*	LOC127292375	4	46649269	46651196	1197	399	Nucleus	IId
*LpWRKY57*	LOC127292440	4	48870931	48906345	3768	1256	Nucleus	III
*LpWRKY58*	LOC127292742	4	62545852	62550169	1233	411	Nucleus	I
*LpWRKY59*	LOC127292768	4	63790559	63794633	957	319	Nucleus	IIc
*LpWRKY60*	LOC127292938	4	71317934	71321349	1167	389	Nucleus	IId
*LpWRKY61*	LOC127293588	4	107809870	107811442	414	138	Nucleus	IIa
*LpWRKY62*	LOC127293765	4	120809075	120812034	1029	343	Nucleus	IId
*LpWRKY63*	LOC127294388	4	163097632	163100562	657	219	Nucleus	III
*LpWRKY64*	LOC127295033	4	215093202	215115137	567	189	Nucleus	IIc
*LpWRKY65*	LOC127332538	4	237699769	237706124	1464	488	Nucleus	I
*LpWRKY66*	LOC127348832	4	268959887	268961473	918	306	Nucleus	III
*LpWRKY67*	LOC127295505	4	278409199	278410996	624	208	Nucleus	III
*LpWRKY68*	LOC127298833	5	19989809	19992831	1053	351	Nucleus	IId
*LpWRKY69*	LOC127299474	5	74232289	74237370	1488	496	Nucleus	I
*LpWRKY70*	LOC127300059	5	139457028	139458823	993	331	Nucleus	III
*LpWRKY71*	LOC127300060	5	139462329	139464099	990	330	Nucleus	III
*LpWRKY72*	LOC127304826	5	139537367	139538788	798	266	Nucleus	III
*LpWRKY73*	LOC127304830	5	139683292	139684525	789	263	Nucleus	III
*LpWRKY74*	LOC127300061	5	139694270	139695670	939	313	Nucleus	III
*LpWRKY75*	LOC127304831	5	139753290	139754571	939	313	Nucleus	III
*LpWRKY76*	LOC127346253	5	175921680	175923506	954	318	Nucleus	III
*LpWRKY77*	LOC127346241	5	176010225	176012002	963	321	Nucleus	III
*LpWRKY78*	LOC127346205	5	176240009	176241871	969	323	Nucleus	III
*LpWRKY79*	LOC127346176	5	176291964	176300532	2007	669	Nucleus	III
*LpWRKY80*	LOC127301565	5	197348969	197350523	978	326	Chloroplast	IIb
*LpWRKY81*	LOC127301563	5	197480727	197482501	804	268	Nucleus	IIb
*LpWRKY82*	LOC127302029	5	222585020	222589236	2070	690	Nucleus	I
*LpWRKY83*	LOC127308096	6	58694863	58696590	1071	357	Nucleus	IIb
*LpWRKY84*	LOC127344942	6	94047980	94049947	1026	342	Nucleus	IIe
*LpWRKY85*	LOC127306880	6	176188843	176191919	708	236	Nucleus	IIc
*LpWRKY86*	LOC127306564	6	193425927	193430478	1650	550	Chloroplast	IIe
*LpWRKY87*	LOC127305985	6	232268093	232270615	1653	551	Nucleus	IIb
*LpWRKY88*	LOC127311307	7	16980175	16984965	1770	590	Nucleus	IIb
*LpWRKY89*	LOC127318134	7	21550346	21556021	1104	368	Nucleus	III
*LpWRKY90*	LOC127313094	7	65861551	65865425	729	243	Nucleus	III
*LpWRKY91*	LOC127316926	7	79615828	79619935	1851	617	Nucleus	I
*LpWRKY92*	LOC127300907	7	151679230	151681492	939	313	Nucleus	III
*LpWRKY93*	LOC127301021	7	169608524	169616726	1563	521	Chloroplast	I
*LpWRKY94*	LOC127314745	7	175638349	175639498	897	299	Nucleus	IId
*LpWRKY95*	LOC127312104	7	231311295	231312867	1347	449	Nucleus	IIe
*LpWRKY96*	LOC127317491	7	316892015	316893584	1014	338	Nucleus	IIb
*LpWRKY97*	LOC127334194	scaffold	302722	304201	969	323	Nucleus	IId

## Data Availability

All data used in this study are available in the Supplementary Materials or from NCBI and plantTFDB.

## Supplementary Materials

The following supporting information can be downloaded at: https://www.mdpi.com/article/10.3390/ijms27209332/s1.
